# Implication of calcium activated RasGRF2 in Annexin A6-mediated breast tumor cell growth and motility

**DOI:** 10.18632/oncotarget.26512

**Published:** 2019-01-04

**Authors:** Diva S. Whalen, Sarrah E. Widatalla, Olga Y. Korolkova, Gladys S. Nangami, Heather K. Beasley, Stephen D. Williams, Carlos Virgous, Brian D. Lehmann, Josiah Ochieng, Amos M. Sakwe

**Affiliations:** ^1^ Department of Biochemistry and Cancer Biology, School of Graduate Studies and Research, Meharry Medical College, Nashville, TN, USA; ^2^ Animal Care Facility, Meharry Medical College, Nashville, TN, USA; ^3^ Department of Biochemistry, Vanderbilt University, Nashville, TN, USA

**Keywords:** annexin A6, breast cancer, RasGRF2, calcium, EGFR

## Abstract

The role of AnxA6 in breast cancer and in particular, the mechanisms underlying its contribution to tumor cell growth and/or motility remain poorly understood. In this study, we established the tumor suppressor function of AnxA6 in triple negative breast cancer (TNBC) cells by showing that loss of AnxA6 is associated with early onset and rapid growth of xenograft TNBC tumors in mice. We also identified the Ca^2+^ activated RasGRF2 as an effector of AnxA6 mediated TNBC cell growth and motility. Activation of Ca^2+^ mobilizing oncogenic receptors such as epidermal growth factor receptor (EGFR) in TNBC cells or pharmacological stimulation of Ca^2+^ influx led to activation, subsequent degradation and altered effector functions of RasGRF2. Inhibition of Ca^2+^ influx or overexpression of AnxA6 blocked the activation/degradation of RasGRF2. We also show that AnxA6 acts as a scaffold for RasGRF2 and Ras proteins and that its interaction with RasGRF2 is modulated by GTP and/or activation of Ras proteins. Meanwhile, down-regulation of RasGRF2 and treatment of cells with the EGFR-targeted tyrosine kinase inhibitor (TKI) lapatinib strongly attenuated the growth of otherwise EGFR-TKI resistant AnxA6 high TNBC cells. These data not only suggest that AnxA6 modulated Ca^2+^ influx and effector functions of RasGRF2 underlie at least in part, the AnxA6 mediated TNBC cell growth and/or motility, but also provide a rationale to target Ras-driven TNBC with EGFR targeted therapies in combination with inhibition of RasGRF2.

## INTRODUCTION

Over the last several years, annexin A6 (AnxA6), a calcium-dependent membrane binding protein, has been shown to play a major role in the differentiation of chondrocytes [1], migration of neural crest cells [2], growth, adhesion and motility of tumor cells [3] and in membrane repair [4, 5]. Some studies suggest that AnxA6 mediates these cellular processes as a multifunctional scaffolding protein for signal transduction molecules [6, 7]. Other reports suggest that it contributes to the remodeling of the actin cytoskeleton [6, 8–10] or modulates the architecture of membrane microdomains such as lipid rafts [11–14], and focal adhesions [3]. Like most annexin family members, the translocation of AnxA6 to cell membranes in response to increased cytosolic Ca^2+^ [15, 16] contributes to the remodeling of membrane microdomains [17] and to maintain the integrity of the plasma membrane [11]. Although the findings thus far remain controversial, membrane lipid rafts appear to be essential for sustained localization of active forms of oncogenic receptors such as the epidermal growth factor receptor (EGFR) [18, 19] and the regulation of Ca^2+^ influx via store-operated Ca^2+^ entry (SOCE) channels [20–22].

Changes in cytosolic Ca^2+^ are known to affect several signal transduction pathways and proteins involved in Ca^2+^ homeostasis [23]. Membrane localization of AnxA6 has been shown to inhibit SOCE [24], while dissociation of AnxA6 from membranes (e.g. during oxidative stress) is accompanied by increased Ca^2+^ influx [25]. The ability of AnxA6 to regulate the tightly controlled Ca^2+^ influx is supported by reports demonstrating that AnxA6 modulates the release Ca^2+^ via interaction with Ca^2+^ channels such as L-type Ca^2+^ channels [26], Na^+^/Ca^2+^ exchangers [26, 27] and sarcoplasmic reticulum Ca^2+^-release channels [28]. Some of these channels are known to cluster within membrane lipid rafts [20–22], but the role of AnxA6 in the activity of these channels and other Ca^2+^ activated proteins remains unclear.

As a member of the annexin family of Ca^2+^ dependent membrane binding proteins, AnxA6 is believed to be a tumor suppressor in many solid tumors [29]. In spite of the intense interest in understanding the role of AnxA6 in cancer, the mechanisms underlying its contribution to triple negative breast cancer (TNBC) cell growth versus tumor cell adhesion and motility have remained elusive. Also evidence supporting its tumor suppressor role in TNBC has thus far been indirect. In this study, we demonstrate that loss of AnxA6 in TNBC cells is associated with early onset and rapid growth of xenograft tumors in mice, thus supporting its role as a tumor suppressor in breast cancer. Our data also suggest that AnxA6 mediated TNBC cell growth and/or motility is dependent on oncogenic receptor stimulated Ca^2+^ entry and effector functions of RasGRF2, and provides a rationale for the use of EGFR/HER2 tyrosine kinase inhibitors (TKIs) in combination with inhibition of RasGRF2 to target Ras driven TNBC tumors.

## RESULTS

### Identification of differentially regulated genes following down-regulation of AnxA6 in invasive breast cancer cells

**Figure 1:**
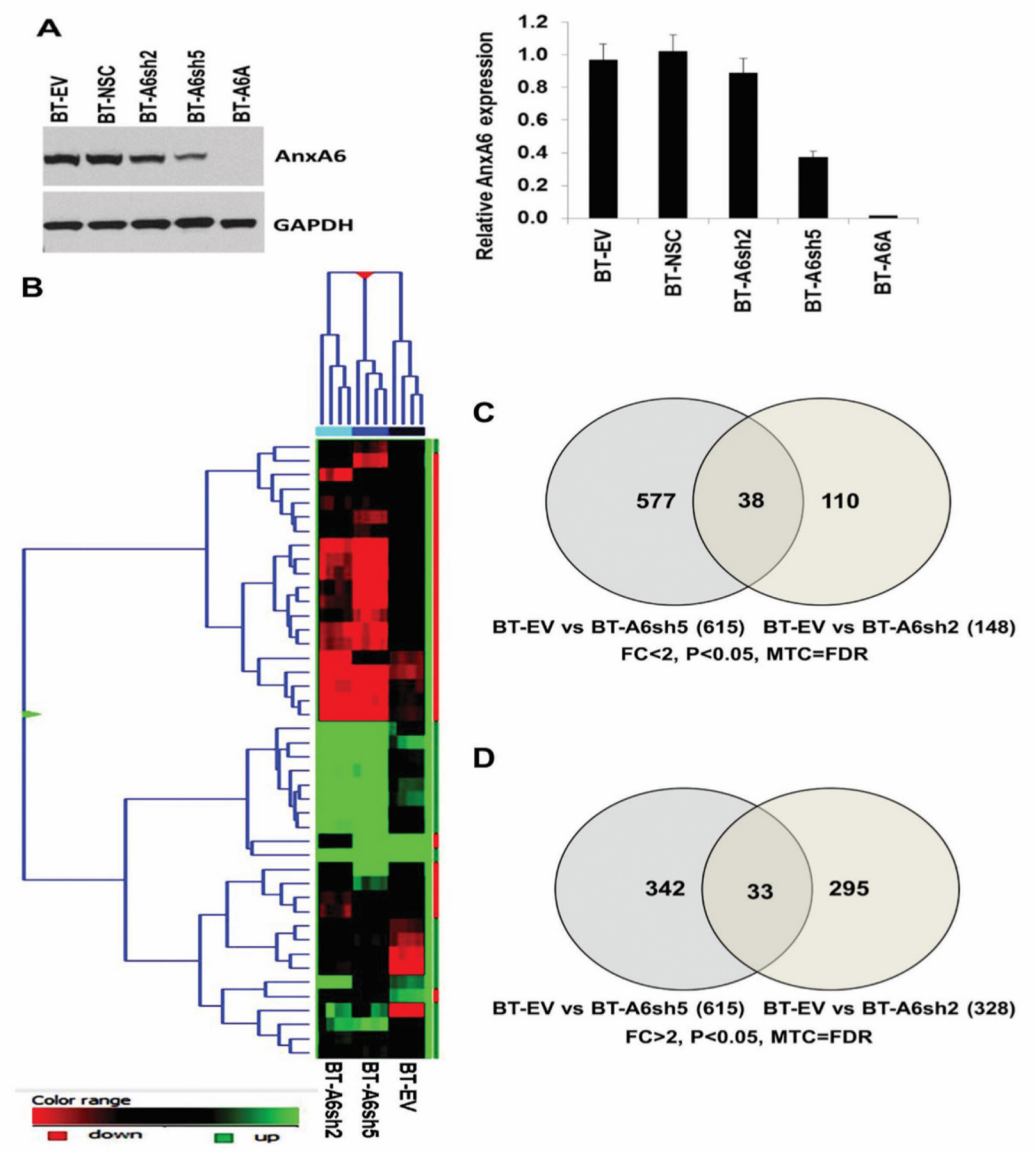
Gene expression profiling of mesenchymal-like BT-549 breast cancer cells following down-regulation of AnxA6 (**A**) Immunoblot showing AnxA6 expression in the indicated subclones of BT-549 cells transfected with shRNAs targeting ANXA6 gene (BT-A6sh2, BT-A6sh5 and BT-A6A), empty vector (BT-EV) or non-targeting control shRNA (BT-NSC). Bar plot shows densitometric quantification of the levels of AnxA6 protein in these subclones relative to the non-targeting control. (**B**) Heatmap depicting the differentially expressed genes in control BT-EV, and in AnxA6 down-regulated BT-A6sh5 and BT-A6sh2 BT-549 derived cell lines. (**C–D**) Venn diagrams displaying the most consistently down-regulated (C) and up-regulated (D) genes following shRNA mediated knockdown of AnxA6 in BT-549 cells.

There is substantial evidence that AnxA6 is a tumor suppressor in many tumor types and that it promotes cell adhesion and motility, however the underlying mechanisms remain poorly understood. To gain a mechanistic understanding of the role of AnxA6 in TNBC, we performed genome-wide gene expression (transcriptome) profiling to identify global gene expression changes following AnxA6 down-regulation. To do this, we used sub-clones of the mesenchymal BT-549 TNBC cell line in which AnxA6 was down-regulated as previously described [3, 30]. As shown in Figure 1A, the BT-549-derived cell lines include BT-549 stably transfected with non-silencing controls (BT-EV/BT-NSC), shRNAs targeting AnxA6 (BT-A6sh2 and BT-A6sh5) [30], and the AnxA6 deficient BT-549-derived cell line designated BT-A6A [3]. Preliminary analysis of differentially expressed transcripts (FDR corrected *p*-value < 0.05) revealed that down-regulation of AnxA6 affected the expression of >500 genes (Supplementary Figure 1) and several hundred more genes were affected following loss of AnxA6 (>98%) as in BT-A6A (data not shown). Hierarchical clustering of differentially expressed genes in control versus AnxA6 down-regulated cells demonstrated similarity among the four technical replicates and separation of control and AnxA6-depleted groups into distinct clusters (Figure 1B). The gene expression data for the AnxA6 depleted cell lines has been deposited in the NCBI's Gene Expression Omnibus (GEO) and is accessible through the accession number GSE72083. Down-regulation of AnxA6 in BT-549 cells resulted in down-regulation of 148 genes in BT-A6sh2 cells and 615 genes in BT-A6sh5 cells (Figure 1C). The analysis also revealed that knockdown of AnxA6 resulted in up-regulation of 328 genes in BT-A6sh2 cells and 375 genes in BT-A6sh5 cells (Figure 1D).

To identify genes that were consistently altered following AnxA6 depletion, we applied a moderated t-test with Benjamini-Hochberg multiple testing correction (MTC = FDR, *p*-value < 0.05, FC>2 or FC<2) on genes that were differentially expressed in both BT-A6sh2 and BT-A6sh5 cell lines. This analysis showed that 38 genes were consistently down-regulated (Figure 1C) and 33 genes were consistently up-regulated (Figure 1D) in both BT-A6sh2 and BT-A6sh5 cells. We also applied more stringent criteria (MTC = FDR, *p*-value < 0.05, FC>2 or FC<2) taking into account the relative differences in AnxA6 depletion (20% in BT-A6sh2 versus >70% in BT-A6sh5 cells). Using these criteria, seven genes (RasGRF2, CDH2, PCHD10, PCDH7, JAG1, FABP4 and MIR4461) were consistently up-regulated following AnxA6 depletion (Supplementary Table 1, shaded area). Five genes (CD24, SLC4A4, METTL7A, APCDD1 and PSG1) were also consistently down-regulated in AnxA6-depleted cells (Supplementary Table 2, shaded area). Among the consistently up-regulated genes, the top four genes including RASGRF2, N-Cadherin (CDH2), and protocadherins 10 (PCDH10) and 7 (PCDH7) encode for Ca^2+^-dependent/activated proteins. Using TaqMan gene expression assays (Life Technologies; Supplementary Table 3), we confirmed that CDH2, FABP4, JAG1 and RASGRF2 were up-regulated (Supplementary Figure 2A) while APCDD1, CD24, METTL7A and SLC4A4 were down-regulated in AnxA6 down-regulated BT-A6sh2 and BT-A6sh5 cells compared to control BT-EV or BT-NSC cells (Supplementary Figure 2B). However, the effect of limited AnxA6 down-regulation appears to be gene specific. Although limited down-regulation of AnxA6 in BT-A6sh2 (<20%) was sufficient to provoke the up-regulation of JAG1 and FABP4, it appears that the reduction in AnxA6 levels was not enough to promote the expression of CDH2 and RasGRF2 (Supplementary Figure 2A). Significant down-regulation of AnxA6 (>70%) in BT-A6sh5 was accompanied by the up-regulation of all these representative genes. The inconsistency in the expression of RasGRF2 and CDH2 in BT-A6sh2 cells with limited AnxA6 knockdown may be due to the relatively high residual levels of AnxA6 and/or insufficient alteration of AnxA6 modulated processes including modulation of Ca^2+^ influx.

Gene Set Enrichment Analysis (GSEA) of all the differentially expressed transcripts revealed that at least 40 cellular signaling pathways were affected by significant AnxA6 knockdown (Supplementary Figure 3). Among these pathways are those previously reported to be associated with changes in AnxA6 expression including cell differentiation [1, 31, 32], cholesterol and lipid homeostasis [12, 33], calcium signaling [24, 33], membrane trafficking [34, 35], EGF/EGFR signaling [3, 25], focal adhesions [3, 25] and regulation of actin cytoskeleton [17, 24]. The pathway analysis also revealed that changes in the expression status of AnxA6 affects cellular processes that are important in cancer progression such as the regulation of cell cycle progression, DNA damage response, energy metabolism, oxidative stress, apoptosis and senescence and autophagy. Of particular interest is the observation that reduced expression of AnxA6 also affected ErbB, TGF-β, insulin, HGF/MET, SCF/Kit, Toll-like receptor, Notch, Wnt and mTOR oncogenic cellular signaling pathways (Supplementary Figure 3).

### Validation of the reciprocal expression of RasGRF2 and AnxA6 in TNBC cells

EGFR is a major oncogene in TNBC cells that potentially plays an important role in RasGRF2 mediated activation of Ras proteins. In an attempt to understand the contribution of AnxA6 in breast tumorigenesis, we first sought to confirm the reciprocal expression of AnxA6 and RasGRF2 at the protein and mRNA levels in TNBC cells and their relationship to EGFR expression levels. Supplementary Figure 4A reveals that the expression of EGFR, AnxA6, and RasGRF2 is cell type specific and potentially, culture condition dependent. It should be noted that the low expression of AnxA6 in SUM149, MDA-MB-468 and HCC70 basal-like TNBC cells [36], is associated with relatively higher expression of RasGRF2. On the contrary, the relatively higher level of AnxA6 in MDA-MB-453, SUM159, SUM185, MDA-MB-157 and MDA-MB-231, which are mesenchymal-like and luminal AR TNBC cells [36], is associated with relatively lower expression of RasGRF2.

**Figure 2:**
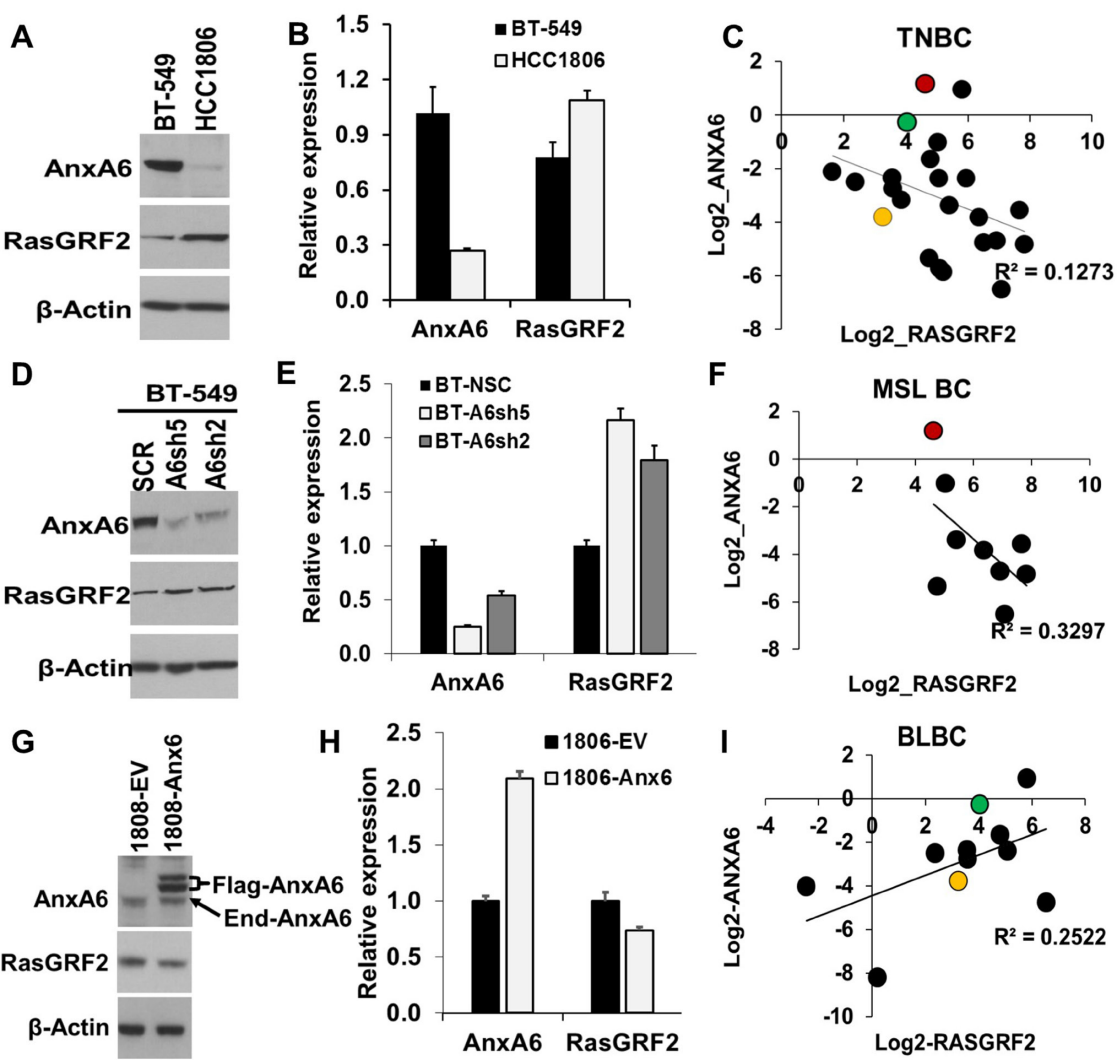
Reciprocal expression of AnxA6 and RasGRF2 in TNBC cells (**A**, **D** and **G**) Representative AnxA6-expressing BT-549 and the AnxA6-low HCC1806 (A), control and AnxA6 down-regulated BT-549 (**D**) or control and AnxA6 up-regulated HCC1806 (G) breast cancer cell lines were grown to 80% confluency then harvested. Equal amounts of whole cell extracts were analyzed by Western blotting using antibodies against AnxA6, RasGRF2 or β-actin. (**B**, **E**, **H**) Densitometric analysis of AnxA6 and RasGRF2 expression in parental BT-549 and HCC1806 cells (B), control and AnxA6 down-regulated BT-549 (E) or control and AnxA6 up-regulated HCC1806 (H) breast cancer cell lines. Bars represent β-actin normalized AnxA6 and RasGRF2 expression from three independent experiments (B and H) or from a representative experiment (E). (**C**, **F**, **I**) Analysis of the relationship between AnxA6 and RasGRF2 mRNA expression in TNBC cells (C), mesenchymal-like TNBC cells (F) and basal-like TNBC cells (I). The mesenchymal-like BT-549 is represented by the red dot, while the basal-like HCC1806 and MDA-MB-468 are represented by the green and yellow dots respectively. TNBC: triple negative breast cancer; MSL BC: mesenchymal-like breast cancer; BLBC: basal-like breast cancer.

To determine the effect of altered expression of AnxA6 on the cellular levels of RasGRF2 in TNBC cells, we selected BT-549 and HCC1806 as model mesenchymal-like and basal-like TNBC cells respectively, but expressing relatively similar levels of EGFR (Supplementary Figure 4A). To minimize culture condition variations, both cell lines were cultured in complete DMEM/F12. Analysis by western blotting revealed that the expression of AnxA6 protein was >3 fold in asynchronously growing BT-549 cells compared to that in HCC1806 cells (Figure 2A and 2B). The expression of RasGRF2 protein on the contrary was a modest ~1.5 fold higher in the basal-like HCC1806 cells compared to that in the mesenchymal-like BT-549 cells (Figure 2A and 2B). To validate the reciprocal expression of AnxA6 and RasGRF2 at the mRNA level, we analyzed RNA-seq gene expression (log2 RPKM) data from 23 TNBC cell lines from a publicly available dataset [37]. This analysis revealed that in most TNBC cell lines, the expression of ANXA6 and RASGRF2 is indeed reciprocal (Figure 2C).

To demonstrate that the cellular levels of RasGRF2 in these cells are dependent on AnxA6 expression status, we next show that RasGRF2 protein levels increased following down-regulation of AnxA6 in the mesenchymal-like BT-549 cells (Figure 2D and 2E). This negative correlation in the expression of these genes was also observed at the mRNA level in nine mesenchymal-like TNBC cell lines [37] (Figure 2F). On the contrary, up-regulation of AnxA6 in the basal-like HCC1806 cells (Figure 2G and 2H) and MDA-MB-468 cells (Supplementary Figure 5) resulted in a decrease in RasGRF2 expression and this was also confirmed in 11 basal-like breast cancer cell lines at the mRNA level (Figure 2I). Figure 2 also revealed that in basal-like TNBC cells in which AnxA6 is least expressed, the correlation between the expression levels of AnxA6 and RasGRF2 is opposite to that in mesenchymal-like TNBC cells in which the expression levels of AnxA6 are relatively high (cf. Figure 2F and 2I). These data validate our microarray analysis of AnxA6 depleted TNBC cells in that the cellular levels of AnxA6 and RasGRF2 are mostly inversely related in TNBC cells. The overall negative correlation of the expression of AnxA6 and RasGRF2 in TNBC cells is consistent with reduced expression of AnxA6 in the majority of TNBCs.

To ascertain whether the reciprocal expression of AnxA6 and RasGRF2 is clinically relevant, we analyzed the effects of low and high expression of these proteins on the survival of basal-like TNBC patients using a publicly available (KM plotter) online survival analysis tool [38]. As shown in Supplementary Figure 3B, the relationship between the expression status of AnxA6 and relapse free survival is inverse to that of RasGRF2. Therefore, highAnxA6/low RasGRF2 is associated with poorer relapse free survival of basal-like TNBC patients compared to those with low AnxA6/high RasGRF2.

### Loss of AnxA6 in invasive breast cancer cells is associated with early onset and rapid xenograft tumor growth

**Figure 3:**
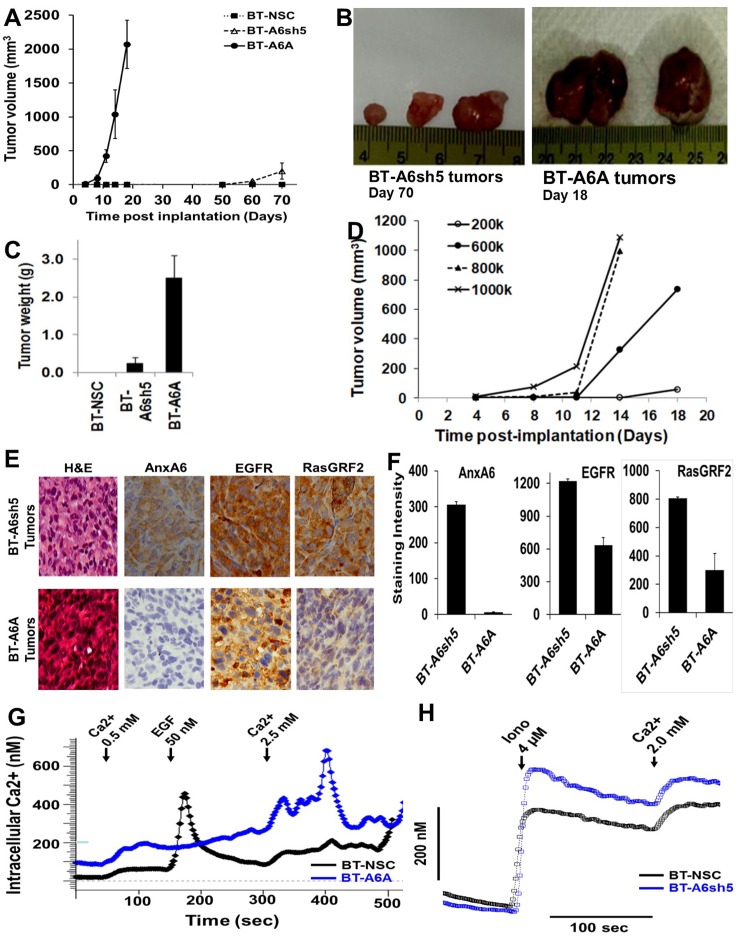
Loss of AnxA6 in invasive breast cancer cells is associated with early onset and rapid xenograft tumor growth (**A**–**C**) Control AnxA6 expressing BT-NSC, AnxA6-depleted BT-A6sh5 and AnxA6 deficient BT-A6A cells (1 × 10^6^/mouse) were implanted into mammary fat pads of 6-7 weeks old Nu/J mice (*n* = 8). The growth of the xenograft tumors was monitored over time (A) and tumor size and weight (B and C) were determined following euthanasia of the tumor bearing mice. (**D**) Nu/J mice were injected with the indicated numbers of AnxA6-deficient BT-A6A cells and tumor volume was monitored as in (A) above. (**E**–**F**) Immunohistochemistry of xenograft tumors. (E) Formalin fixed, paraffin embedded thin sections of xenograft tumor tissues derived from AnxA6 down-regulated BT-A6sh5 and AnxA6 deficient BT-A6A cells were stained with antibodies against AnxA6, EGFR and RasGRF2 as well as with hematoxylin-eosin. (F) Immunostained tumor tissue sections were digitally scanned and quantified using the Tissue IA software (Leica Microsystems). ^**^indicates *p* < 0.01. **G**–**H**) Intracellular Ca^2+^ spectrofluorimetry. Cell suspensions were loaded with fura-2 AM and changes in intracellular Ca^2+^ concentration were recorded in real time using the Hitachi F2500 spectrofluorimeter. Representative traces showing activation of store-operated Ca^2+^ influx by treatment of BT-NSC and BT-A6A cells with EGF followed by addition of Ca^2+^ (H) or by treatment of BT-NSC and BT-A6sh5 with ionomycin followed by addition of Ca^2+^ (G).

Although AnxA6 has been demonstrated to be a tumor suppressor in several tumor types [3, 29, 39–42], evidence supporting its role in breast cancer has thus far remained indirect. Here, we selected the poorly tumorigenic BT-549 TNBC cells (tumor latency 2–3 months) [43] to test the hypothesis that reduced expression of AnxA6 is associated with tumor growth. To do this, we implanted equal numbers of control (BT-NSC), AnxA6 down-regulated (BT-A6sh5) and AnxA6-deficient (BT-A6A) subclones of BT-549 cells (Figure 1A) into mammary fat pads of Nu/J athymic nude mice. Consistent with previous reports [43] the AnxA6 expressing BT-NSC control cells failed to form tumors in this nude mouse model over the course of the experiment (Figure 3A and 3C). However, significant down-regulation (>70 %) of AnxA6 in the BT-A6sh5 cells led to latent (~50 days) xenograft tumor formation (Figure 3A) while implantation of the same number of AnxA6 deficient BT-A6A cells led to both early tumor onset (within 7 days) and rapid tumor growth that necessitated humane euthanasia of the mice within 18 days (Figure 3A–3C). To validate the early xenograft tumor onset of AnxA6 deficient BT-A6A cells, we demonstrate that injection of fewer cells delayed tumor onset in a dose dependent manner followed by rapid growth of the ensuing xenograft tumors (Figure 3D).

Given that reduced expression of AnxA6 is associated with increased expression of the Ca^2+^-activated RasGRF2 (Figure 2), we speculated that increased levels of RasGRF2 may drive the rapid growth of the xenograft tumors following AnxA6 down-regulation or loss in BT-549 cells. To test this, we stained the tumor tissues derived from the BT-A6sh5 cells and AnxA6-deficient BT-A6A cells by immunohistochemistry. As expected, AnxA6 was barely detected in xenograft tumors derived from AnxA6 deficient BT-A6A cells compared to that in tumors derived from BT-A6sh5 cells (Figure 3E and 3F). Consistent with our recent report [26], the expression of EGFR was also decreased by 2-fold in tumors derived from AnxA6 deficient cells compared to that in tumors derived from AnxA6 down-regulated BT-A6sh5 cells (Figure 3E and 3F). Surprisingly, the expression level of RasGRF2 in tumors from AnxA6-deficient cells, was >2-fold lower than that in tumors derived from AnxA6-depleted BT-A6sh5 cells (Figure 3E and 3F). Since the activity of RasGRF2 is Ca^2+^ dependent and activation of RasGRF2 has been shown to be accompanied by its down-regulation [44], we speculated that reduced expression or loss of AnxA6 may be associated with higher cytosolic Ca^2+^ levels and/or deregulated Ca^2+^ influx. To test this, we assessed the intracellular Ca^2+^ dynamics by spectrofluorimetry. We show that control AnxA6 expressing cells responded to EGF treatment with release of Ca^2+^ from intracellular stores and this was accompanied by store operated Ca^2+^ entry in the presence of up to 2.5 mM Ca^2+^. On the contrary, AnxA6 deficient BT-A6A cells apparently lost their responsiveness to EGF and showed deregulated Ca^2+^ entry in the presence of 2.5 mM Ca^2+^ and consequently higher cytosolic Ca^2+^ levels (Figure 3G). We next showed that following ionomycin treatment, intracellular Ca^2+^ levels were higher in AnxA6 depleted BT-A6sh5 cells compared to AnxA6 expressing control cells (Figure 3H). Meanwhile, modest AnxA6 down-regulation was associated with reduced responsiveness to EGF but did not significantly alter the Ca^2+^ influx dynamics compared to control AnxA6 expressing cells (data not shown). Together with data in Figure 2, these data suggest that the reciprocal expression of AnxA6 and RasGRF2 in TNBC cells is dependent at least in part, on AnxA6 regulated plasma membrane permeability to extracellular Ca^2+^.

### Up-regulation of AnxA6 is associated with increased Cdc42 activity and cell motility but attenuated xenograft tumor growth

**Figure 4:**
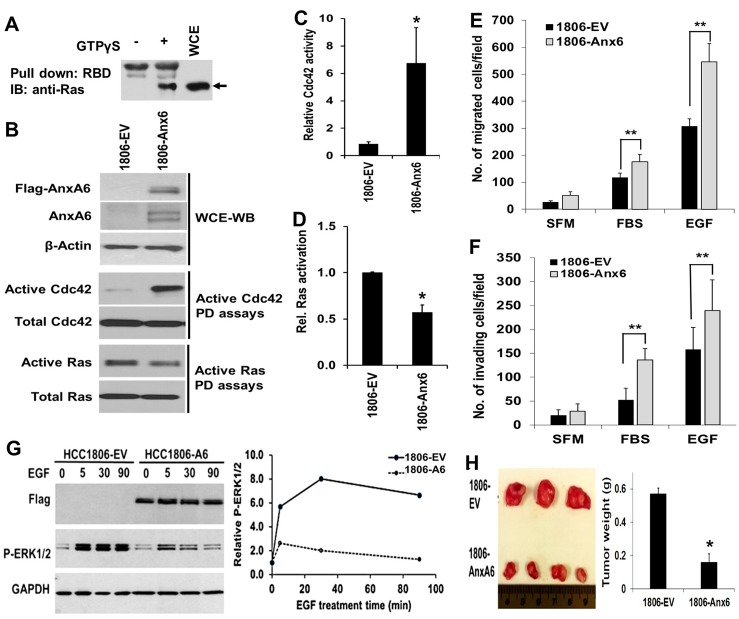
Up-regulation of AnxA6 in the basal-like HCC1806 breast cancer cells is associated with decreased xenograft tumor growth (**A**–**D**) Control (1806-EV) and HCC1806 expressing flag-tagged AnxA6 (1806-Anx6) cells were grown until 80% confluent and harvested by scrapping. WCE were prepared as described in materials and methods, and equal amounts of protein were used in GTPase activation assays. A) Typical Ras activation assay in the presence and absence of GTP-γS. B) Analysis of AnxA6 expression (WCE-WB) and pull-down assays for GTP-γS bound Ras (B) or GTP-γS bound Cdc42 (B) using GST-Raf-1 binding domain (RDB) and GST-PAK-1 binding domain (PDB) respectively by Western blot analysis. C and D) Densitometric analysis of GTP bound Ras or GTP bound Cdc42. Bars represent GTP bound Ras (C) or GTP-bound Cdc42 (D) levels relative to the total levels of the respective GTPases in the control 1806-EV and 1806-AnxA6 cells from at least two independent determinations. (**E**–**F**) Migration and invasion assays. Serum-starved cells were plated in Boyden chambers with (E) or without (F) a thin layer of Matrigel in serum free medium. Complete medium containing 10% FBS or EGF in SFM were used as chemoattractants. (**G**) Control (1806-EV) and HCC1806 expressing flag-tagged AnxA6 (1806-Anx6) cells were grown until 70% confluent, then serum starved for 24 h and incubated with EGF 50 ng/ml in HBSS containing 0.5 mM Mg^2+^, and 0.5 mM Ca^2+^ for the indicated time points. Cells were harvested and the activation of ERK1/2 analyzed by western blotting. (**H**) Control and flag-tagged AnxA6 expressing HCC1806 cells were injected into mammary fat pads of Nu/J mice and the tumor size and weight were determined as described in Figure 3. ^*^p < 0.05; ^**^p < 0.01.

We previously showed that down-regulation of AnxA6 in TNBC cells was associated with increased anchorage independent cell growth [3] but on the contrary, inhibited cell motility [26]. It has also been shown that RasGRF2 promotes cell growth through its RasGEF activity and inhibits cell motility via inhibition of Cdc42 [25]. These reports and data in Figures 2 and 3, prompted us to speculate that RasGRF2 may be an effector for AnxA6 mediated tumor cell growth and motility. To test this we carried out GTPase activation assays (Figure 4A) to determine whether altered AnxA6 expression affected the effector functions of RasGRF2 i.e. activation of Ras proteins to promote tumor cell growth and inhibition of Cdc42 to inhibit tumor cell motility. We demonstrate that exogenous expression of AnxA6 was associated with increased (>6 fold) Cdc42 activity (Figure 4B and 4C) but inhibited Ras activity by ~2-fold (Figure 4B and 4D). Consistent with the increase in Cdc42 activity, overexpression of AnxA6 in these cells also promoted EGF-stimulated invasion (Figure 4E) and cell migration (Figure 4F). Furthermore, expression of AnxA6 in HCC1806 cells strongly attenuated EGF induced activation of ERK1/2 compared to the control cells (Figure 4G). To ascertain that the increase in AnxA6 levels which is accompanied by relatively lower RasGRF2 levels as demonstrated in Figure 2, was associated with decreased tumor growth, we implanted empty vector transfected HCC1806 cells (1806-EV) and flag-tagged AnxA6-transfected HCC1806 (1806-Anx6) cells (2 × 10^6^ cells/mouse) into mammary fat pads of Nu/J mice. Analysis of tumor growth revealed that up-regulation of AnxA6 inhibited the growth of xenograft tumors, consistent with its tumor suppressor function (Figure 4H). Together these data not only support the tumor suppressor function of AnxA6 but also suggest that the effector functions of RasGRF2, cell growth via Ras activation and inhibition of cell motility via Cdc42, are important in AnxA6 modulated tumor growth and cell motility.

### AnxA6-modulated Ca^2+^ influx and subsequent activation/degradation of RasGRF2 underlies the differential expression of RasGRF2 in breast cancer cells

**Figure 5:**
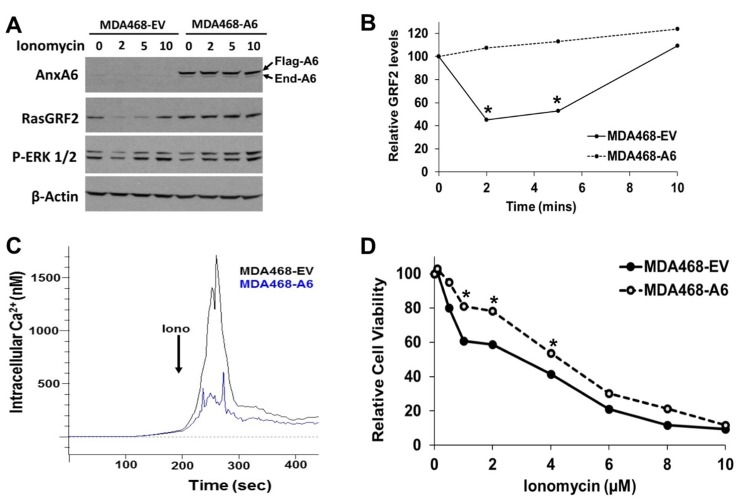
Up-regulation of AnxA6 in AnxA6 low TNBC cells inhibited ionomycin-induced Ca^2+^ surge and RasGRF2 down-regulation (**A**) MDA-MB-468 cells were transfected with plasmids encoding the empty vector or flag-tagged AnxA6 were grown to 70% confluency, then treated with ionomycin (2 μM) for the indicated time points. Cells were harvested and WCE were used for western blotting and probed with antibodies against AnxA6, RasGRF2, p-ERK1/2 and β-actin (loading control). (**B**) Densitometric analysis of the effects of ionomycin induced Ca^2+^ surge on the cellular levels of RasGRF2 in control AnxA6 low versus AnxA6 overexpressing TNBC cells from a representative experiment. (**C**) Determination of the effects of AnxA6 expression on ionomycin induced increase in intracellular Ca^2+^ in Fura-2 loaded control or AnxA6 up-regulated MDA-MB-468 cells by spectrofluorimetry. (**D**) Effects of ionomycin on the proliferation of control and AnxA6 overexpressing MDA-MB-468 cells. Cell proliferation was determined from quadruplicate samples using the PrestoBlue cell viability assay reagent. ^*^indicates *p* < 0.05.

Previous reports have demonstrated that AnxA6 inhibits store-operated Ca^2+^ influx [24], that the activity of RasGRF2 is Ca^2+^ dependent [45, 46] and that ionomycin induced surge in intracellular Ca^2+^ leads to down-regulation of RasGRF2 [44]. To provide a mechanistic insight into the reciprocal expression of AnxA6 and RasGRF2, we expressed flag-tagged AnxA6 in EGFR-amplified MDA-MB-468 cells and assessed the effects of ionomycin induced Ca^2+^ surge on the cellular levels of RasGRF2. We show that ionomycin treatment was associated with rapid but transient (within 2 min) down-regulation of RasGRF2 in control AnxA6 low MDA-MB-468 cells. On the contrary, RasGRF2 was stabilized in AnxA6 expressing cells following treatment with ionomycin (Figure 5A and 5B). To ascertain that the rapid down-regulation of RasGRF2 in AnxA6 low cells was due to ionomycin induced sudden increase in cytosolic Ca^2+^, we measured the surge in intracellular Ca^2+^ levels following treatment of fura-2 AM loaded control and AnxA6 expressing cells with ionomycin. This analysis revealed that up-regulation of AnxA6 in MDA-MB-468 cells strongly inhibited the surge in intracellular Ca^2+^ compared to that in control cells (Figure 5C). We next show that the viability of AnxA6 low control cells was significantly lower than that of AnxA6 expressing cells when cultured in the presence of lower less toxic concentrations (0.5–4 μM) of ionomycin (Figure 5D).

**Figure 6:**
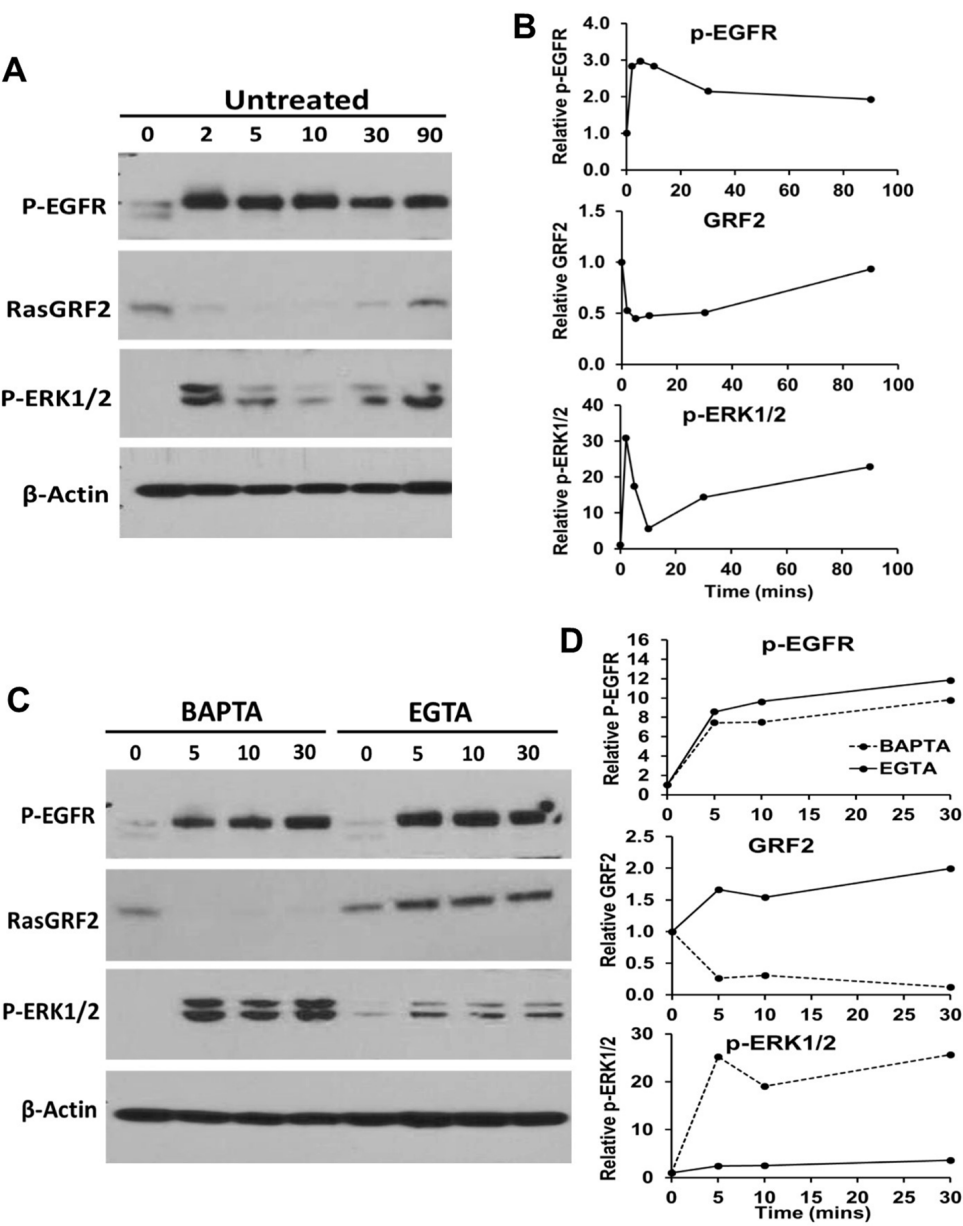
Down-regulation of RasGRF2 requires activation of EGFR and Ca^2+^ influx from extracellular milieu (**A**) MDA-MB-468 cells were treated with or without EGF (50 ng/ml) following a time course and the cellular levels of activated EGFR (phospho-Y1068), RasGRF2, phospho-ERK1/2 and β-actin (loading control) assessed by western blotting; (**B**) Densitometric analysis of the cellular levels of these proteins over the 90 min time course for a representative experiment. (**C**) Cells were pretreated with BAPTA or EGTA for 1 h, then stimulated with EGF (50 ng/ml) in the presence of the respective Ca^2+^ chelators for the indicated times. The expression of phospho-EGFR, RasGRF2 and Phospho-ERK1/2 was assessed by western blotting. (**D**) Densitometric analysis of the cellular levels of these proteins over the 30 min time course for a representative experiment. Experiments were repeated at least three times with similar results.

Since AnxA6 has been shown to inhibit store-operated Ca^2+^ entry [24], we next examined the possibility that Ca^2+^ influx provoked by activation of Ca^2+^ mobilizing oncogenes such as EGFR in TNBC cells could lead to RasGRF2 down-regulation. MDA-MB-468 cells were treated with EGF (50 ng/ml) in the presence or absence of the intracellular Ca^2+^ chelator BAPTA or the extracellular Ca^2+^ chelator EGTA. Detection of phospho-EGFR (Y1068) by western blotting revealed an initial rapid activation followed by a sustained activation of the receptor (Figure 6A and 6B). The initial activation of EGFR was accompanied by a correspondingly rapid decrease in the cellular levels of RasGRF2 (within 2 min of EGF treatment) followed by recovery to basal levels by 90 min. Consistent with the activation of EGFR and the accompanying down-regulation of RasGRF2, activation of ERK1/2 also occurred within 2 min followed by a lower but sustained activation beyond 10 min of EGF treatment (Figure 6A and 6B). This is consistent with our previous report in which we demonstrated that in cells expressing low levels of AnxA6 such as MDA-MB-468 cells, activated EGFR is rapidly internalized and the activation of ERK1/2 is transient and barely detected 10 min post-EGF treatment [30]. These data suggest that the initial phase of EGFR activation corresponds to the presence of activated EGFR on the cell surface, the stimulation of Ca^2+^ influx and activation of RasGRF2 and robust activation of ERK1/2. The sustained phase on the other hand, corresponds to the internalized EGFR during which the activity of EGFR and consequently stimulation of Ca^2+^ influx are greatly diminished and RasGRF2 and activated ERK1/2 levels return to near basal levels.

The rapid decrease in RasGRF2 levels and the acute activation of EGFR suggested that rapid down-regulation of RasGRF2 resulted from its Ca^2+^ dependent activation and as previously demonstrated, proteasomal degradation [44]. To prove this we pretreated MDA-MB-468 cells with BAPTA or EGTA followed by treatment with EGF. Figure 6C and 6D show that chelation of intracellular Ca^2+^ with BAPTA followed by EGFR activation did not block the down-regulation of RasGRF2 and led to a sustained activation of ERK1/2. Conversely, the cellular levels of RasGRF2 were stabilized following chelation of extracellular Ca^2+^ with EGTA and this was associated with a correspondingly strong inhibition of ERK1/2 activation. These data suggest that oncogenic receptor (e.g. EGFR) stimulated and AnxA6 modulated Ca^2+^ influx is at least in part, the basis for the reciprocal expression levels of AnxA6 and RasGRF2 in TNBC cells.

### Subcellular localization and potential interaction of AnxA6 with RasGRF2

**Figure 7:**
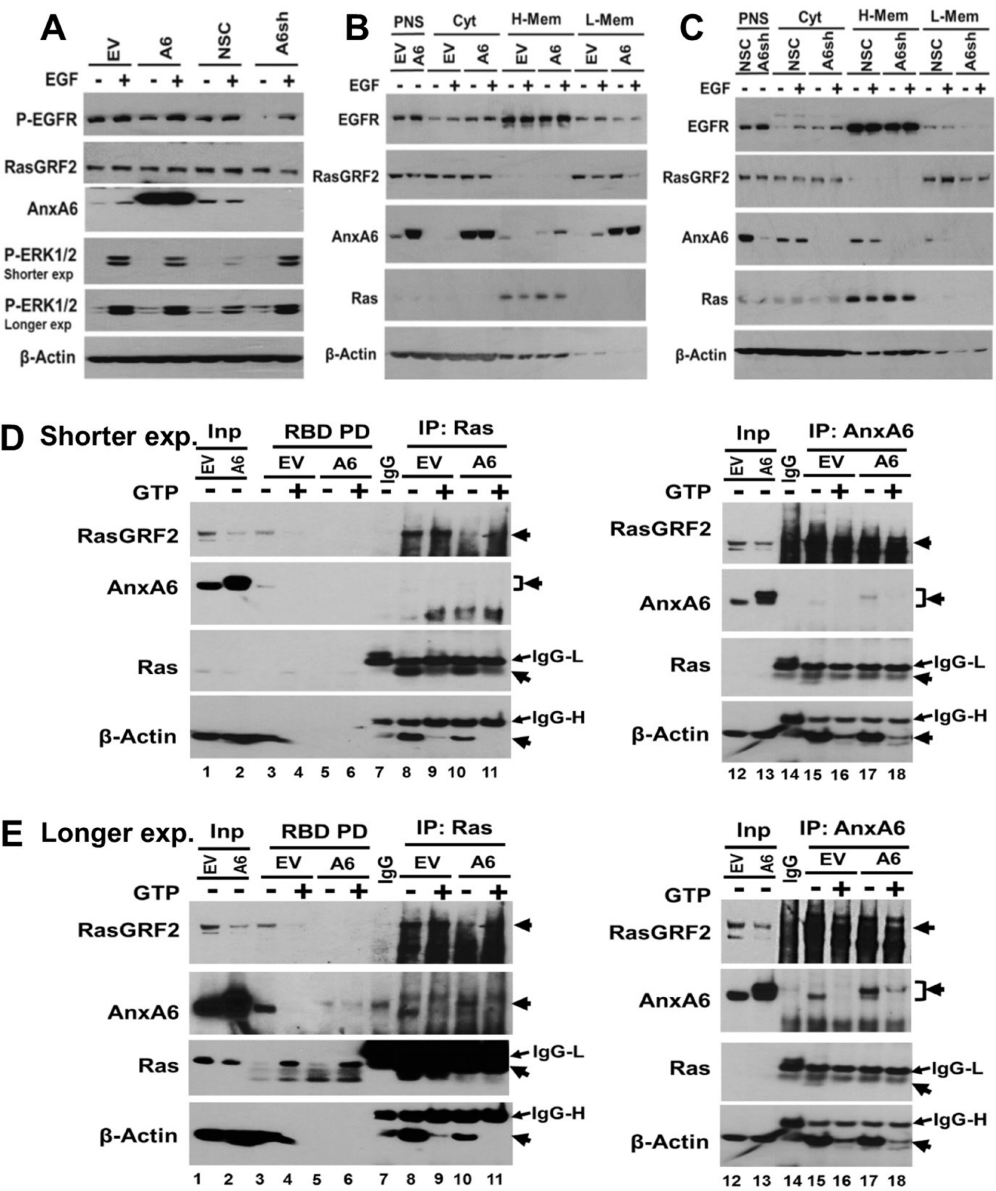
Subcellular localization and interaction of AnxA6 with RasGRF2 (**A–C**) The indicated cell lines were grown to 70% confluency, treated with or without EGF, harvested by scrapping in ice-cold PBS and fractionated by differential velocity centrifugation as described in methods. The post-nuclear supernatants (A) or the various fractions from control and AnxA6 up-regulated MDA-MB-468 cells (B) and control and AnxA6 down-regulated MDA-MB-468 cells (C) were analyzed by western blotting. (**D**–**E**) Detection of AnxA6, RasGRF2, Ras proteins and β-actin in GST-Raf-1 binding domain (RBD) pull downs and co-IPs. Equal amounts of whole cell extracts prepared as described in Figure 4A and B from control and AnxA6 overexpressing MDA-MB-468 cells, were used in Ras-GTP pull down assays (RBD) or immunoprecipitation using antibodies to pan-Ras (IP: Ras) or AnxA6 (IP: AnxA6). GST-RBD bound proteins as well as protein A/G bound immune complexes were analyzed by western blotting using antibodies against Ras, RasGRF2, AnxA6 and β-actin. Blots were revealed by ECL. Shown are shorter (D) and longer (E) exposures. EV: empty vector transfected; A6: flag-AnxA6 transfected; NSC: no silencing control transfected; and A6sh: shRNA targeting AnxA6 transfected MDA-MB-468 cells. PNS: Post-nuclear supernatant; H-Mem: Heavy membranes; L-Mem: lighter membranes; Cyt: cytosol.

Our data thus far suggest that modulation of Ca^2+^ influx by AnxA6 and Ca^2+^ dependent activation followed by degradation of RasGRF2 underlies the reciprocal expression of these proteins in TNBC cells. As previously demonstrated, AnxA6 is a scaffolding protein for signaling effectors such as PKC-α and p120GAP [6, 7]. To determine whether the cellular localization of RasGRF2 is altered by AnxA6 expression and/or EGFR activation, we analyzed the expression of AnxA6, RasGRF2, Ras proteins and b-actin in MDA-MB-468 cells in which AnxA6 was either up-regulated or down-regulated. Cells were serum starved overnight and treated with EGF for 5 min, then the post-nuclear supernatant was fractionated into cytosolic, 21,000 × g (heavy) membranes and >100,000 × g (lighter) membranes. We first show that EGF treatment of MDA-MB-468 cells led to ERK1/2 activation in the post-nuclear supernatants (Figure 7A). We next show that Ras proteins and RasGRF2 are spatially segregated (Figure 7B and 7C). As expected Ras proteins are predominantly associated with heavier membranes (H-Mem) while RasGRF2 is not only predominantly cytosolic (Cyt) but also associated with lighter membranes (L-Mem). Altered AnxA6 expression did not alter the levels of RasGRF2 in the cytosolic pool while EGF treatment of MDA-MB-468 cells more conspicuously led to attenuated levels of RasGRF2 in the lighter membrane pool and that this was dependent on AnxA6 and EGF treatment (Figure 7B, L-Mem). Although AnxA6 is predominantly cytosolic in control NSC cells (Figure 7C), overexpression of AnxA6 revealed equally abundant levels in lighter membrane pool (Figure 7B). EGF treatment on the other hand, led to AnxA6 translocation to both lighter and heavier membranes (Figure 7B and 7C). Meanwhile, like Ras proteins, EGFR is predominantly detected in the heavy membranes (Figure 7B and 7C), suggesting that the heavy membranes are plasma membranes while the lighter membranes are endosomal. Detection of ectopically expressed AnxA6 in the lighter endosomal membranes is consistent with previous studies [12, 47].

To determine whether AnxA6 may also act as a scaffolding protein for RasGRF2, we first used the GST-Raf-1 binding domain (RBD) to pull-down Ras in the presence or absence of GTP and then assessed the presence of AnxA6 and/or RasGRF2 in the complex. Consistent with data in Figure 2, overexpression of AnxA6 led to lower whole cellular levels of RasGRF2 (Figure 7D and 7E, lanes 1 and 2). We next show that in AnxA6 low EV control cells, AnxA6 and RasGRF2 appear to be in a stable complex with Ras-GDP. However, in the presence of GTP or following activation of Ras (Ras-GTP), the level of RasGRF2 noticeably decreased and AnxA6 is undetectable in the complex even after a relatively long exposure (Figure 7E, lanes 3 and 4). In AnxA6 overexpressing cells, AnxA6 was detected in the complex but RasGRF2 was undetected in the presence of either Ras-GDP or Ras-GTP (Figure 7D, lanes 5 and 6). To test this further, we carried out co-immunoprecipitaition (co-IP) using antibodies to pan-Ras (Figure 7D and 7E, lanes 7–11). This confirmed that in AnxA6 low EV control cells, RasGRF2 is in complex with inactive Ras proteins (Figure 7D, lanes 8 and 9) and that upon Ras activation or AnxA6 overexpression, RasGRF2 in the complex was noticeably reduced (Figure 7D, lanes 10 and 11). Meanwhile, β-actin was also strongly detected in the co-IPs using antibodies to either Ras proteins or AnxA6 and the interaction of RasGRF2, Ras and β-actin appears to be more stable in AnxA6 low control cells than in AnxA6 overexpressing cells and destabilized by GTP or Ras activation (Figure 7D and 7E, lanes 8–11). A similar co-IP with antibodies against AnxA6 revealed similar results in that the immune complexes contained Ras proteins and b-actin and that AnxA6 and both Ras proteins and β-actin were strongly reduced in the presence of GTP. On the other hand, RasGRF2 in the anti-AnxA6 immune complexes was detected as a smear (Figure 7D and 7E, lanes 15–18). Together this suggests that activation of Ras proteins and/or AnxA6 upregulation is associated with reduced levels and potentially attenuated interaction of RasGRF2 with its substrate Ras proteins. While further studies are warranted to determine whether AnxA6 and RasGRF2 interact directly or indirectly via β-actin, it is possible that AnxA6 acts as a scaffold for RasGRF2 and that the interaction occurs either in the cytosol or in microsomal membranes.

### Down-regulation of RasGRF2 and inhibition of EGFR strongly inhibits the growth of TNBC cells

**Figure 8:**
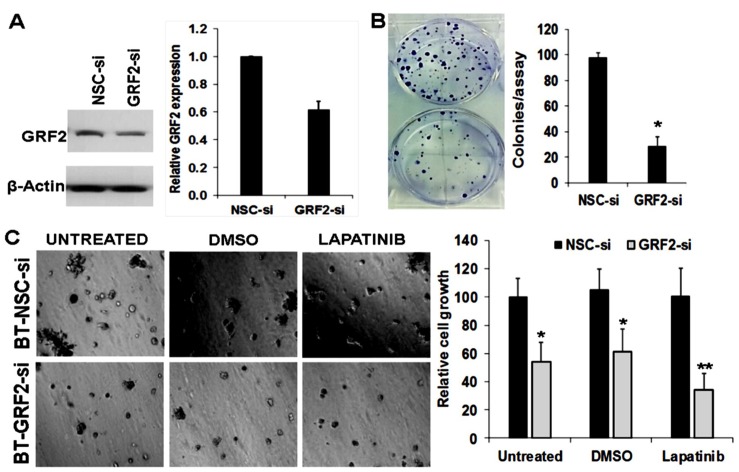
Down-regulation of RasGRF2 and inhibition of EGFR potently blocks the growth of TNBC cells (**A**) RNAi mediated down-regulation of RasGRF2 in AnxA6 expressing BT-549 cells. Cells were transfected with pools of 4 control non silencing siRNA (NSC-si) or 4 siRNAs targeting the coding sequence of RasGRF2 (GRF2-si). Cells were harvested and the down-regulation of RasGRF2 assessed by western blotting (left panel) and densitometric analysis of the protein band (right panel). (**B**) Clonogenic assays of control and RasGRF2 down-regulated cells. After 5 days in culture, control NSC-si and GRF2-si transfected cells were stained using crystal violet (0.5% in 1% acetic acid) and the growth of the cells was assessed by the number of colonies. (**C**) Control NSC-si and GRF2-si transfected cells were grown in growth factor reduced Matrigel 3D cultures for up to 7 days in the presence or absence of lapatinib (2 μM). Cell growth/viability was assessed using the PrestoBlue cell viability reagent and images of colonies were captured digitally. ^*^indicates *p* < 0.05; ^**^indicates *p* < 0.01.

As a scaffolding protein, AnxA6 cannot be easily targeted for therapeutic purposes but effector proteins such as the as yet unknown AnxA6 specific SOCE channels or RasGRF2 appear to be attractive targets. We have also shown that AnxA6 high cells are more resistant to EGFR targeted tyrosine kinase inhibitors [30]. Figures 4, 5 and Supplementary Figure 5C suggest a role for Ca^2+^ mobilizing oncogenic cell surface receptor tyrosine kinases (e.g. EGFR) and GPCRs affected by AnxA6 down-regulation (Supplementary Figure 3) in the activation of RasGRF2. Based on these data and in the absence of pharmacological inhibitors of RasGRF2, we hypothesized that combined inhibition of EGFR and down-regulation of RasGRF2 may be more effective in inhibiting the growth of AnxA6 high TNBC cells. To test this, BT-549 cells were transfected with control non-targeting or a siRNA pool consisting of four distinct siRNAs directed against RasGRF2 followed by analysis of cell growth and viability in 3D cultures with or without treatment with lapatinib. We show that down-regulation of RasGRF2 in these cells (Figure 8A) resulted in fewer and smaller colonies compared to the control siRNA transfected cells (Figure 8B). Interestingly, down-regulation of RasGRF2 and inhibition of EGFR by treatment of the cells with lapatinib led to a greater decrease in cell growth (Figure 8C). This suggests that inhibition of EGFR is not sufficient to block the growth of TNBC cells and that combined inhibition of RasGRF2 and EGFR may be exploited as a viable option to block the growth and /or motility of TNBC cells.

## DISCUSSION

The role of AnxA6 in breast cancer and in particular, the mechanisms underlying its contribution to tumor cell growth and/or motility remain poorly understood. In this study, we identified and validated RasGRF2 as one of the genes that is significantly up-regulated following down-regulation of AnxA6 in TNBC cells. We also established the hitherto undemonstrated tumor suppressor function of AnxA6 in TNBC cells, demonstrated that the reciprocal expression of AnxA6 and RasGRF2 is dependent on the modulation of Ca^2+^ influx by AnxA6 and that the contribution of AnxA6 in tumor cell growth and motility is at least in part, mediated by the effector functions of RasGRF2 i.e. activation of Ras proteins and inhibition of Cdc42 respectively. Besides modulation of Ca^2+^ influx, we also show that AnxA6 presumably in concert with F-actin, acts as a scaffold for RasGRF2 and other components involved in the activation of Ras proteins. Interestingly, inhibition of EGFR and down-regulation of RasGRF2 strongly attenuated the growth of otherwise EGFR-TKI resistant AnxA6 high TNBC cells. Given that activation of RasGRF2 by Ca^2+^ mobilizing oncogenes constitutes an important axis for Ras activation by several RTKs and GPCRs, this study provides a strong rationale to target these hard-to treat breast tumors with EGFR targeted therapies in combination with inhibition of RasGRF2.

The finding that reduced expression or loss of AnxA6, in the otherwise poorly tumorigenic BT-549 cells [43], is necessary for the onset and rapid growth of xenograft tumors in mice is quite intriguing. Our data reveal that down-regulation of AnxA6 not only affected several Ca^2+^ mobilizing receptor tyrosine kinase and G protein couple receptor signaling pathways [48, 49] but also the WNT, Notch and other signaling pathways known to promote cell proliferation, survival, migration, and cell fate determination. The further decrease in the expression of CD24 and up-regulation of JAG1 following AnxA6 down-regulation suggest that reduced expression or loss of AnxA6 is associated with poorly differentiated, highly proliferative tumor cells. This phenotype is consistent with a previous report showing that loss of AnxA6 delayed the differentiation of chondrocytes [1]. This phenotype is also consistent with our previous findings that low AnxA6 expression was associated with poor distant metastasis-free and overall survival of basal-like breast cancer patients and not those with other molecular subtypes or breast cancer patients in general [30]. In agreement with other studies [50] and although EGFR is a major oncogene in TNBC tumors, the rapid growth of AnxA6-low or AnxA6-deficient tumors appears to be EGFR-independent. The dysregulation of Ca^2+^ influx in cells with significantly reduced expression or loss of AnxA6 suggests that the growth of these tumors may be partly driven by high cytosolic Ca^2+^ dependent mechanisms.

A major outcome of this study is the finding that the cellular levels of AnxA6 are inversely related to those of RasGRF2. As previously demonstrated [50], cells expressing relatively high levels of AnxA6 are more motile but grow relatively poorly. On the other hand, high RasGRF2 expressing cells have been shown to promote cell growth but inhibit tumor cell motility [50]. Together, this supports the model that potentially aggressive TNBC cells are AnxA6 low but RasGRF2 high while potentially motile TNBC cells are AnxA6 high but RasGRF2 low. The reciprocal expression of AnxA6 and RasGRF2 is supported by three biochemical assays including altered expression of AnxA6 in three TNBC cell lines and assessment of basal levels of the proteins; by demonstrating the requirement for a surge in intracellular Ca^2+^ as the underlying basis for RasGRF2 degradation; by assessing the effects of altered expression of AnxA6 on effector functions of RasGRF2 through Ras/Cdc42 GTPase activation assays; and by assessing the expression of these proteins in several TNBC cell lines at the protein and mRNA levels. Even though this was replicated in some TNBC cell lines, it was not the case for a number of TNBC cells, thus underscoring the heterogeneity of TNBC cells lines. It is also worth noting that the reciprocal expression of these genes may be influenced by the distinct requirements for the growth of TNBC cells *in vitro*. It is also possible that genes such as RasGRF2 and/or pathways that are dependent on AnxA6 regulated Ca^2+^ entry, require significant up or down-regulation of AnxA6 levels for quantifiable effects. This may explain why mild (<50%) down-regulation or residual levels of AnxA6 only slightly or did not replicate the reciprocal expression of RasGRF2 and CDH2 but AnxA6 dependently affected the expression of other genes such as JAG1 and FABP4.

Given that several cellular processes are dependent on cytosolic Ca^2+^ dynamics, it is not surprising that reduced expression of AnxA6 affects several signaling pathways and cellular processes. A surge in cytosolic Ca^2+^ often results from exposure of cells to drugs that stimulate Ca^2+^ uptake (ionophores) or following activation of cell surface oncogenic RTKs or GPCRs. One of the unifying outcomes of stimulated Ca^2+^ influx is the potential for the Ca^2+^ surge to activate RasGRF2 [45, 46] and consequently its effector functions in cell growth as a Ras protein specific GEF and in cell motility by inhibiting the activity of Cdc42 [51]. Our data suggest that the primary source of Ca^2+^ for activation/down-regulation of RasGRF2 is extracellular. Since Ca^2+^ influx is modulated by AnxA6 and RasGRF2 promotes cell growth and inhibits cell motility, our data suggest that high RasGRF2/low AnxA6 may define rapid tumor growth and poor invasiveness while low RasGRF2/high AnxA6 may be associated with invasive but slow growing tumors. This is supported by the stabilization of RasGRF2 following chelation of extracellular Ca^2+^ by treatment of cells with EGTA or by inhibition of Ca^2+^ uptake following overexpression of AnxA6. Additionally, and although loss of AnxA6 and the accompanying dysregulation of Ca^2+^ influx supported rapid growth of xenograft tumors, overexpression of AnxA6 not only supported increased cell motility but also attenuated xenograft tumor growth.

AnxA6 has been shown to act as a scaffold for activated PKC as well as p120GAP [6]. Our finding that AnxA6 is in the same complex with RasGRF2, Ras proteins and actin is consistent with its scaffolding functions. It remains to be determined whether AnxA6 directly or indirectly interacts with RasGRF2. However, it is possible that AnxA6 facilitates the translocation of RasGRF2 to heavy membrane anchored Ras proteins based on its Ca^2+^ dependent membrane translocation [6]. It is also possible that AnxA6 facilitates the nucleotide exchange activity of RasGRF2 since AnxA6 has been reported to bind GTP [52]. The diminished cellular levels of RasGRF2 in AnxA6 expressing cells and the associated decrease in the activation of Ras and ERK1/2 as well as reduced tumor growth are consistent with the inhibition of the growth of AnxA6 expressing tumor cells. However, it is hard to rule out other potential mechanisms including the previously described AnxA6/p120GAP interaction [53]. Our data suggest that the motility of AnxA6 expressing cells may be mediated via RasGRF2 by inhibition of Cdc42. An indication that cell motility is inhibited by relatively higher levels of RasGRF2 is also supported by the report showing that up-regulation of RasGRF2 following down-regulation of β-arrestin1 attenuated cell migration and invasion and that this occurred via inhibition of Rac [54]. Although other mechanisms may be involved, our study supports the notion that the tumor associated functions of AnxA6 hinge on its ability to modulate the activity of Ca^2+^ influx channels and to aggregate factors that mediate the activation of small GTPases including Ras, Cdc42 and Rac.

Although far from being exhaustive, our data support the model that in AnxA6 expressing TNBC cells, activation of Ca^2+^ mobilizing oncogenes such as EGFR stimulate SOCE and the ensuing activation and degradation of RasGRF2 will lead to overall diminished cellular levels of this RasGEF. The rapid decrease in RasGRF2 levels will relieve its inhibition on Cdc42 and allow active Cdc42 to inhibit the EGFR ubiquitin ligase c-Cbl [55] and therefore, block proteasome mediated degradation of the activated receptor [56]. This, as previously demonstrated results in the stabilization of activated EGFR on the cell surface [30] and thus, the increased cell motility. On the contrary, in AnxA6 low TNBC cells, activated EGFR is rapidly endocytosed but the endosome-associated activated receptors signal poorly as previously demonstrated [57, 58]. The rapid internalization of activated receptors will lead to transient and reduced SOCE, stabilization of RasGRF2 and inhibition of Cdc42. Ultimately, this will not only allow c-Cbl to be active, but will also lead to c-Cbl mediated ubiquitinylation, degradation of the internalized activated EGFR [55], and reduced cell motility. Meanwhile, the stabilized RasGRF2 in these cells may be activated by other Ca^2+^-mobilizing oncogenic receptors [59] to promote tumor cell growth. This model is supported by the reduced EGFR staining in AnxA6 deficient xenograft tumors, deregulated Ca^2+^ influx and aggressive tumor growth following significant down-regulation or loss of AnxA6, reduced RasGRF2 levels following stimulated Ca^2+^ influx and the stabilization of GRF2 levels following inhibition of Ca^2+^ entry or up-regulation of AnxA6. This model also support the notion that combined inhibition of EGFR and RasGRF2 may be a viable option to not only block the growth of TNBC cells but also their motility.

In summary, reduced expression of AnxA6 appears to be a critical event in breast carcinogenesis, as loss of AnxA6 is associated with early onset and rapid tumor growth. Although the cellular cues that provoke down-regulation of AnxA6 in solid rumors and the role of other RasGEFs remain unknown, this study underscores the importance of AnxA6 modulated Ca^2+^ influx and the effector functions of RasGRF2 in AnxA6 mediated tumor growth and/or motility. This study also provides a rationale for the use of EGFR/HER2 tyrosine kinase inhibitors in combination with inhibition of RasGRF2 to target Ras driven triple negative breast tumors.

## MATERIALS AND METHODS

### Cell lines and cell culture

BT-549 and HCC1806 breast cancer cells (ATCC, East Rutherford, NJ) were maintained in DMEM/F12 supplemented with 10% fetal bovine serum (FBS,), L-glutamine (2 mM), 100 units/ml penicillin, and 50 units/ml streptomycin (Invitrogen, Carlsbad, CA). MDA-MB-468 were also obtained from the ATCC and were maintained in Leibovitz's L15 medium (Invitrogen) supplemented with 10 mM NaHCO_3_, 5% FBS, penicillin (100 units/ml), and streptomycin (50 units/ml). BT-549 breast cancer cell lines stably transfected with empty vector (BT-EV), non-silencing shRNA (BT-NSC) or AnxA6 targeting shRNAs designated BT-A6sh2 and BT-A6sh5 respectively, as well as HCC1806 cells stably transfected with AnxA6 cDNA (1806-Anx6) or empty vector control (1806-EV) were generated as recently described [30]. AnxA6 was also stably expressed in MDA-MB-468 cells which express amplified levels of EGFR as previously described [30]. AnxA6 deficient BT-549 (BT-A6A) cells have also been described previously [3]. Control and RasGRF2 targeting siRNA pools (Dharmacon, Cat # M-024516-0) were purchased from GE-Dharmacon. Where necessary serum-free medium (SFM) comprised the respective media supplemented with 0.5% FBS (Atlanta Biologicals, Atlanta, GA). These cell lines were cultured in a humidified 95% air and 5% CO_2_ incubator at 37° C and media were changed every 2–3 days. Except otherwise indicated, treatment of cells with calcium chelators, EGFR-targeted tyrosine kinase inhibitors (TKIs) or EGF was carried out in Ca^2+^ /Mg^2+^-free Hank's balanced salt solution (HBSS, Invitrogen) supplemented with 0.5 mM CaCl_2_ and 0.5 mM MgCl_2_.

### Gene expression profiling and data analyses

Total RNA was extracted from control and AnxA6 down-regulated BT-549 (*n* = 4/construct) cells cultured in complete medium for 48 h using the RNeasy kit (Qiagen, Valencia, CA). Samples selected for gene expression arrays, were those with high RNA integrity number (>7). These were processed and loaded on the Affymetrix human gene 2.0ST cartridge array (Santa Clara, CA) and hybridized overnight in an Affymetrix Model 645 Hybridization Oven. After hybridization, the cartridge arrays were washed, and stained per standard Affymetrix protocols using an Affymetrix Fluidics Station 450. The arrays were then scanned in an Affymetrix 7G plus scanner and the resulting data were analyzed using the Affymetrix Expression Console v1.2 and a *Robust Multi-array* Average (RMA) normalization algorithm producing log base 2 results. Differential gene expression was assessed between replicate groups (*n* = 4) using a moderated t-test with Benjamini-Hochberg multiple testing correction (MTC), with significance determined by adjusted *p*-value < 0.05 and absolute value fold change (FC) of >2 for up-regulated genes or <2 for down-regulated genes. Hierarchical clustering was performed using GeneSpring on both averaged and non-averaged normalized expression values. The gene expression data for the AnxA6 depleted cell lines has been deposited in the NCBI's Gene Expression Omnibus (GEO) and is accessible through the accession number GSE72083.

### Reverse transcriptase and semi-quantitative real-time PCR (RT-PCR)

For the validation of the identified genes, total RNA was isolated from AnxA6 down-regulated and control cells as described above. First-strand cDNA synthesis was performed using the iScript cDNA synthesis kit following the protocol provided by the manufacturer (Bio-Rad, Hercules, CA). This was followed by semi-quantitative real time PCR using individual TaqMan assays and universal TaqMan gene expression master mix (Life Technologies, Grand Island, NY). The expression of GAPDH or β-actin was used as an internal standard.

### Whole cell extracts and Immunoblotting

The indicated control mammary epithelial and TNBC cell lines cultured as recommended by the suppliers were harvested by the Pietenpol lab at Vanderbilt Ingram Cancer Center. Whole cell extracts (WCE) from these cell lines and the model mesenchymal-like and basal-like cell lines were prepared in radioimmunoprecipitation assay (RIPA) buffer (50 mM Tris-HCl, pH 7.4, 1% NP-40, 0.1% sodium deoxycholate, 150 mM NaCl, 1 mM EDTA) and freshly added protease and phosphatase inhibitors. Immunoblotting was performed as previously described [3] and the blots were revealed by enhanced chemiluminescence (Perkin Elmer) and where applicable, quantified using the NIH ImageJ software.

### Subcellular fractionation

Subcellular fractionation was performed as previously described [60] with some modifications. Briefly, cells were harvested by scrapping in ice-cold PBS, then disrupted by Dounce homogenization and the post-nuclear supernatant (PNS) isolated by centrifugation at 1500 × g for 5 min at 4° C. The PNS was then centrifuges at 21,000 × g to isolate heavy membranes and the resulting supernatant centrifuged at >100,000 × g for 2 h at 4° C to isolate the lighter (microsomal) membranes. The isolated membranes were then solubilized in RIPA buffer followed by centrifugation at 10,000 × g for 10 min at 4° C.

### Immunohistochemistry

Formalin-fixed paraffin-embedded tissue sections (8 μm thickness) prepared from xenograft tumors from the AnxA6 down-regulated BT-A6sh5 or the AnxA6-deficient BT-A6A cells were stained as previously described [3] using the following primary antibodies: RasGRF2 (Abcam, Cambridge, MA), AnxA6 (Santa Cruz Biotechnology Inc., Santa Cruz, CA); EGFR (Cell Signaling Technology, Danvers, MA). For quantification of the immunostaining, slides were digitally scanned at the Digital Histology Shared Resource at Vanderbilt University Medical Center. The stained tissue areas were digitally demarcated and the staining intensity analyzed by using the Tissue IA software (Leica Microsystems).

### Ras and Cdc42 activation and immunoprecipitation assays

Asynchronously growing cells were harvested by scrapping in ice-cold PBS. Cell lysis and the levels of GTP-bound Ras or GTP-bound Cdc42 were determined using the Ras and Rac1/Cdc42 activation kits respectively as described by the manufacturer (Millipore, Temecula, CA). Active (GTPγS-bound) Ras or Cdc42 in the pull-down assays as well total levels of these GTPases were visualized by western blotting using the respective antibodies and quantified using the NIH ImageJ software. For immunoprecipitation, equal amounts of protein extracts prepared as described above were incubated overnight at 4° C with antibodies against pan-Ras (Millipore) or AnxA6 and protein A/G plus agarose beads. The beads were washed and bound proteins analyzed by western blotting as described above.

### Cell proliferation and motility assays

Cell growth assays were performed using PrestoBlue cell viability/proliferation reagent (Life Technologies) while growth in 3D cultures was carried out by using growth factor reduced a Matrigel respectively. Invasion and migration assays were performed in Boyden chambers with or without a thin layer of Matrigel as previously described [3]. Complete medium containing 10% FBS or serum free medium with or without EGF (50 ng/ml) were used as chemo-attractants and the number invaded/migrated cells were microscopically counted after staining with 0.5% crystal violet.

### Measurement of intracellular calcium

Intracellular calcium was measured as previously described [61] using Fura-2 AM (1 μM final concentration) loaded cell suspensions and a Hitachi F2500 spectrofluorimeter.

### Animal experiments

All experiments were conducted in accordance with general guidelines in the protocol approved by the Institutional Animal Care and Use Committee (IACUC) at Meharry Medical College. The facilities and laboratory animals programs at Meharry Medical College are accredited by the Association for the Assessment and Accreditation of Laboratory Animal Care. The IACUC ensured that animal related experiments adhered to the National Institutes of Health (NIH) guidelines for the humane care and use of laboratory animals. The 5 to 6 week old female Nu/J athymic nude mice were obtained from Jackson Laboratories. Suspensions of control, AnxA6 down-regulated and AnxA6 deficient BT-549 cells (1 × 10^6^ viable cells/100 μl)or control and AnxA6 expressing HCC1806 cells (2 × 10^6^ viable cells/100 μl) were subcutaneously injected into the mammary fat pads of the mice (*n* = 8). Tumor growth was monitored by bi-weekly measurement of tumor volume. Mice were euthanized at the end of the experiment or when tumors were ~2,000 mm^3^. Tumor tissues were processed for H&E and immunohistochemical staining according to standard procedures.

### Statistical analyses

The difference in the expression of RasGRF2, AnxA6 as well as differences in the activation of Ras and CDC42 were analyzed using the Student's paired t-test for samples with unequal variance. Analyses of tumor growth characteristics (tumor volume or weight) were performed using two-way ANOVA. A *p* value < 0.05 was considered to be statistically significant.

## SUPPLEMENTARY MATERIALS FIGURES AND TABLES


