# Supplementary material for: Implication of calcium activated RasGRF2 in Annexin A6-mediated breast tumor cell growth and motility
Source: Oncotarget. 2019 Jan 4;10(2):133–51. doi: 10.18632/oncotarget.26512 (PMC6349432; doi:10.18632/oncotarget.26512)
Supplement: Supplementary file 1 [file oncotarget-10-133-s001.pdf]

Implication of calcium activated RasGRF2 in Annexin A6-mediated breast tumor cell growth and motility

SUPPLEMENTARY MATERIALS

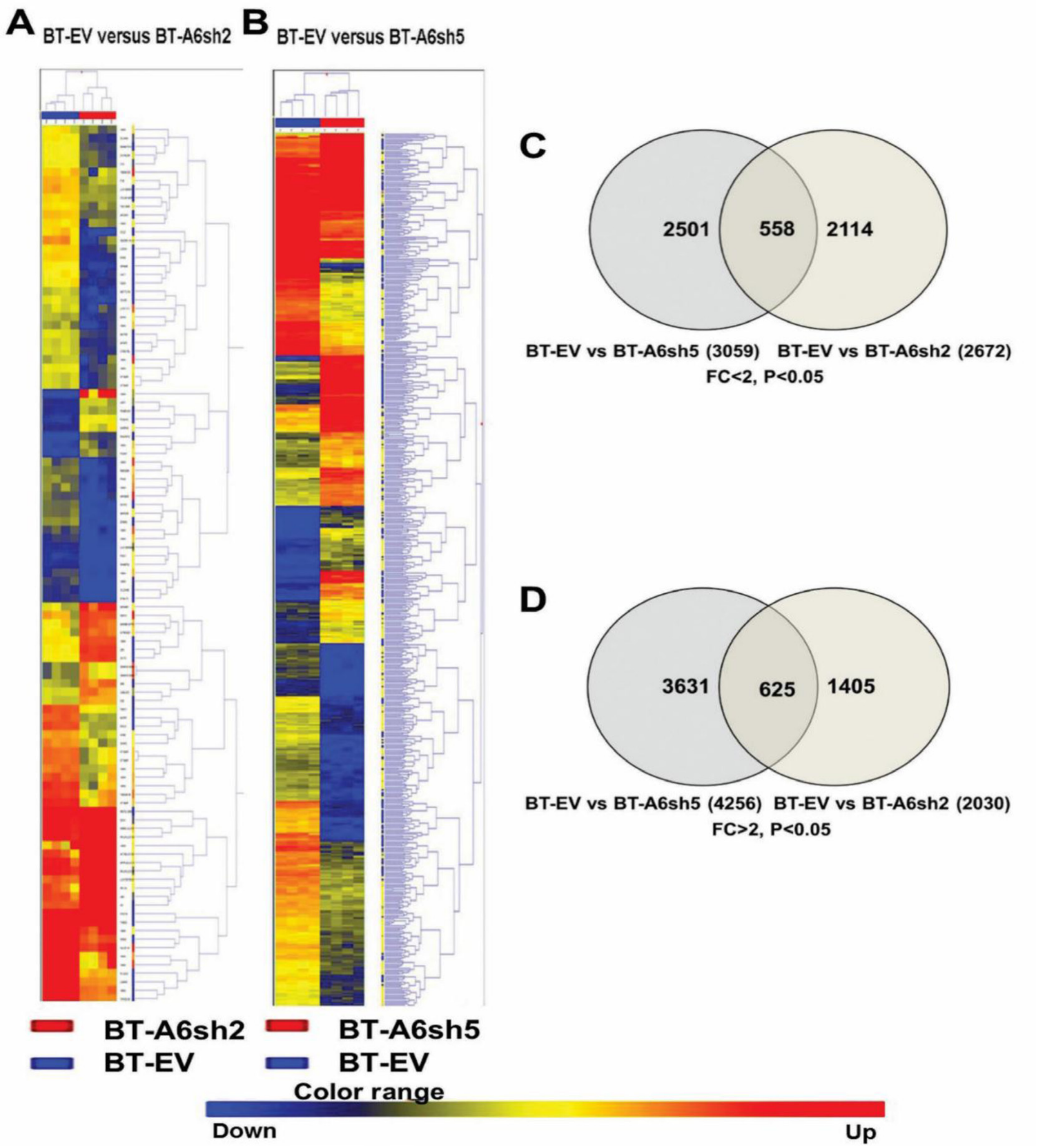

Supplemntary Figure 1: Heatmap of differentially expressed genes following AnxA6 down-regulation in mesenchymal-like BT-549 breast cancer cells. Hierarchical clustering of differentially expressed genes in the control BT-EV versus AnxA6 down regulated BT-A6sh2 (**A**) and in the control BT-EV versus AnxA6 down regulated BT-A6sh5 cells (**B–D**) Differentially down-regulated genes (C) and up-regulated genes (D) following RNAi mediated knockdown of AnxA6 in BT-549 cells. Note that the number of affected genes is greater with greater AnxA6 down-regulation.

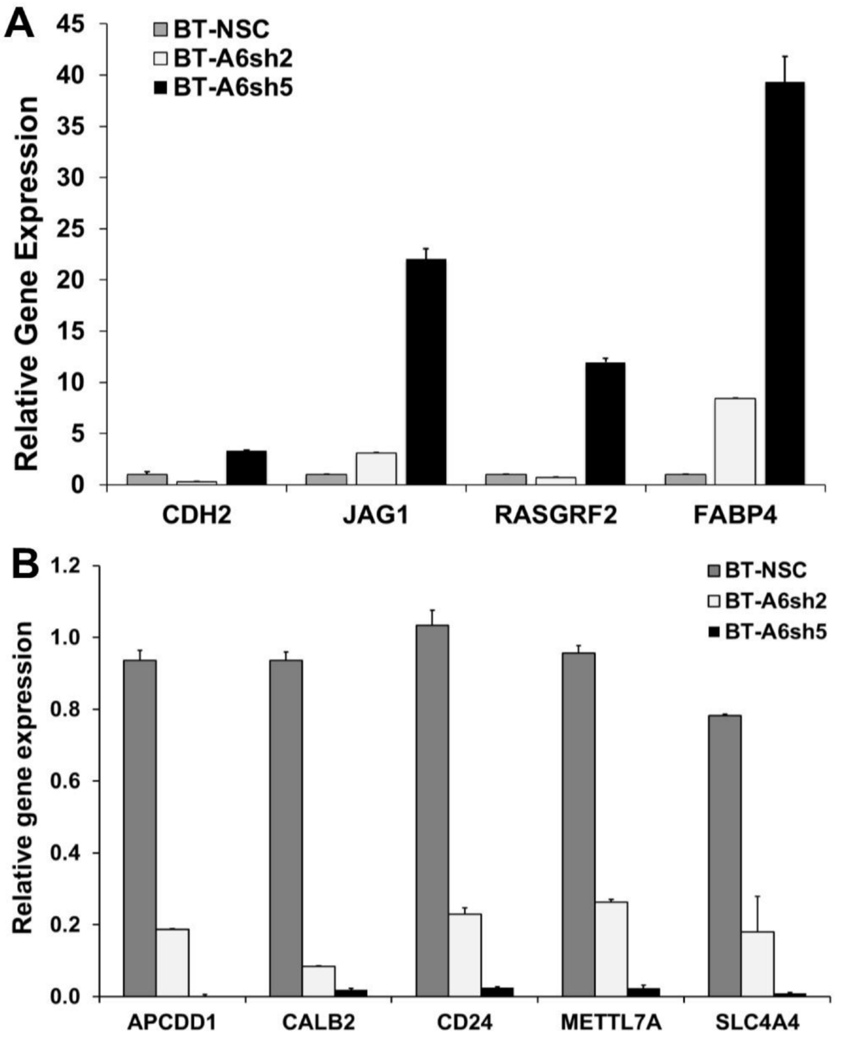

Supplementary Figure 2: Validation of AnxA6-dependent differentially expressed genes by semi-quantitative RT-PCR. Total RNA was isolated from the indicated BT-549 derived cell line and equal amount of total RNA was used for semi-quantitative RT-PCR using Taqman assays for the indicated up regulated genes (**A**) and down-regulated genes (**B**). Each bar represents the expression of the indicated genes relative to the level in the control BT-EV cells from three independent determinations.

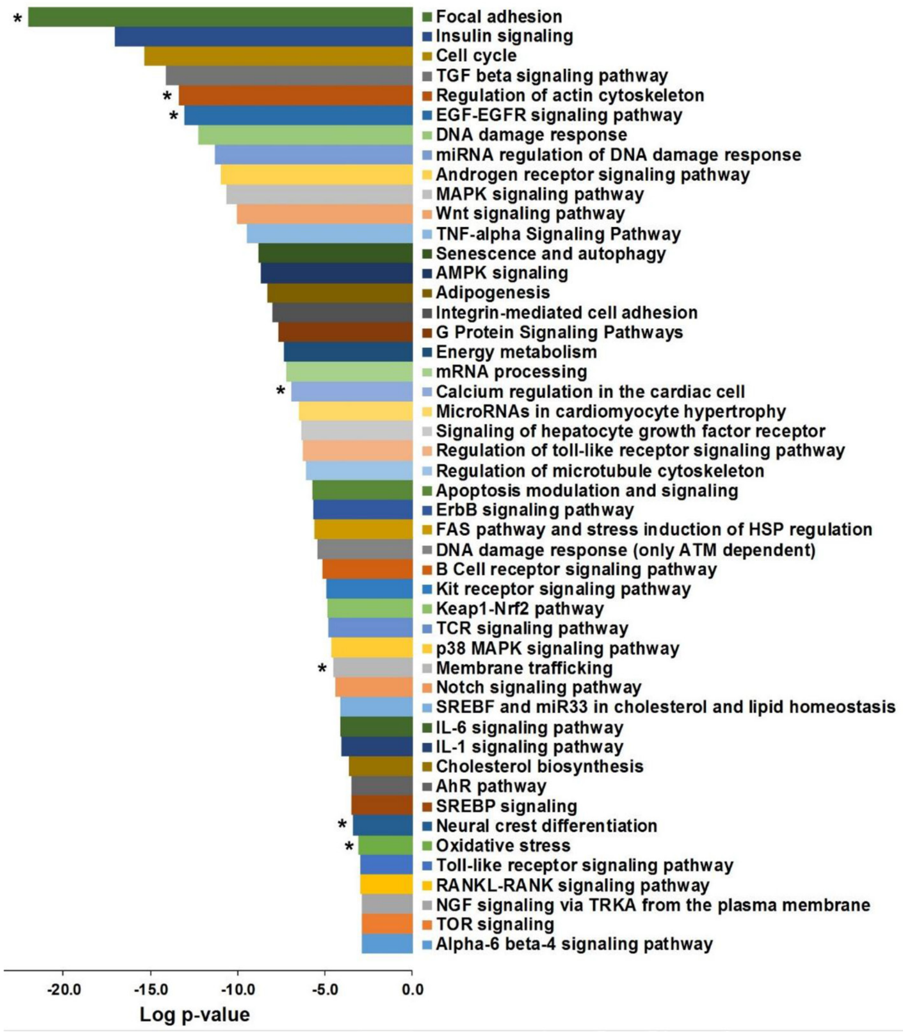

Supplementary Figure 3: Pathways affected by reduced expression of the tumor suppressor AnxA6. *denote pathways already reported to be associated with altered expression of AnxA6.

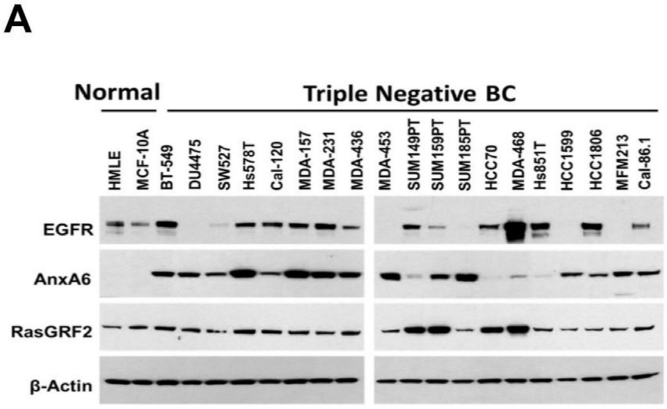

	EGFR	ANXA6	RASGRF2
HMLE	1.00	1.00	1.00
MCF10A	0.62	1.13	1.37
BT-549	1.28	5.19	1.45
DU4475	0.30	5.91	1.70
SW527	0.31	3.78	1.01
Hs578T	0.74	9.05	1.46
Cal-120	0.96	5.05	1.46
MDA-157	0.85	9.65	1.17
MDA-231	1.16	9.50	1.11
MDA-436	0.67	8.19	1.54
MDA-453	0.28	10.32	1.39
SUM149	1.01	1.80	2.95
SUM159	0.36	6.45	3.17
SUM185	0.20	13.22	1.16
HCC70	0.75	1.57	2.14
MDA-468	3.55	2.49	3.09
Hs851T	1.69	0.90	1.11
HCC1599	0.15	7.05	0.98
HCC1806	1.77	3.30	0.77
MF213	0.11	5.00	0.81
Cal-86.1	0.54	3.66	1.00

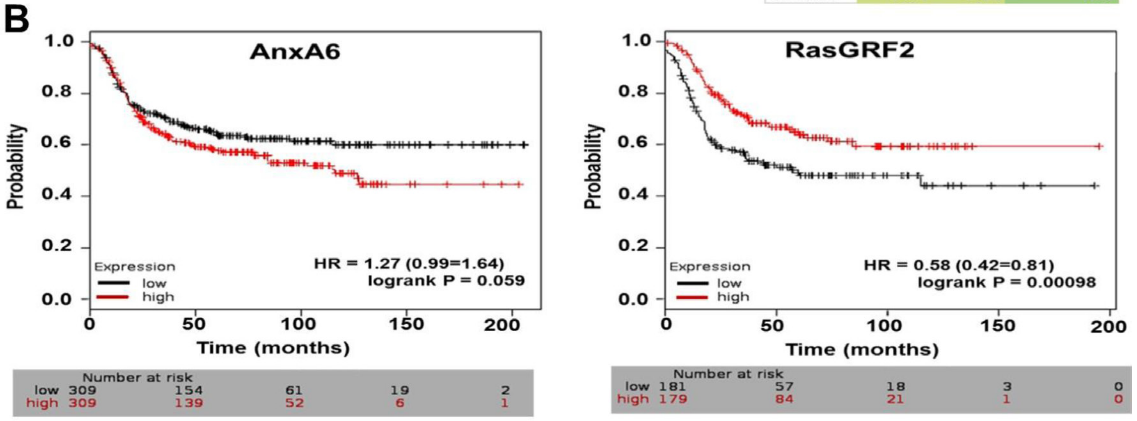

Supplementary Figure 4: Reciprocal expression of AnxA6 and RasGRF2 in TNBC cell lines. (**A**) Cell pellets from the indicated control mammary epithelial and TNBC cell lines grown as recommended by the suppliers were prepared by the Pietenpol lab at Vanderbilt Ingram Cancer Center. These were lysed in radioimmunoprecipitation buffer, and immunoblotting was performed as previously described in materials and methods. The blots were revealed by enhanced chemiluminescence (Perkin Elmer) and quantified using the NIH ImageJ software. The color coded values for each protein are β-actin normalized expression and relative to primary HMLE cells. (**B**) Survival analyses of patients with high and low AnxA6-expressing basal-like breast cancers. Kaplan Meier plots showing the relationship between relapse-free survival of basal-like breast cancer patients and high or low AnxA6 and RasGRF2 expression. The Kaplan-Meier plots and the number of patients at risk at given time points are indicated for low AnxA6 or RasGRF2 expression status (black) and for high AnxA6 or RasGRF2 expression status (red).

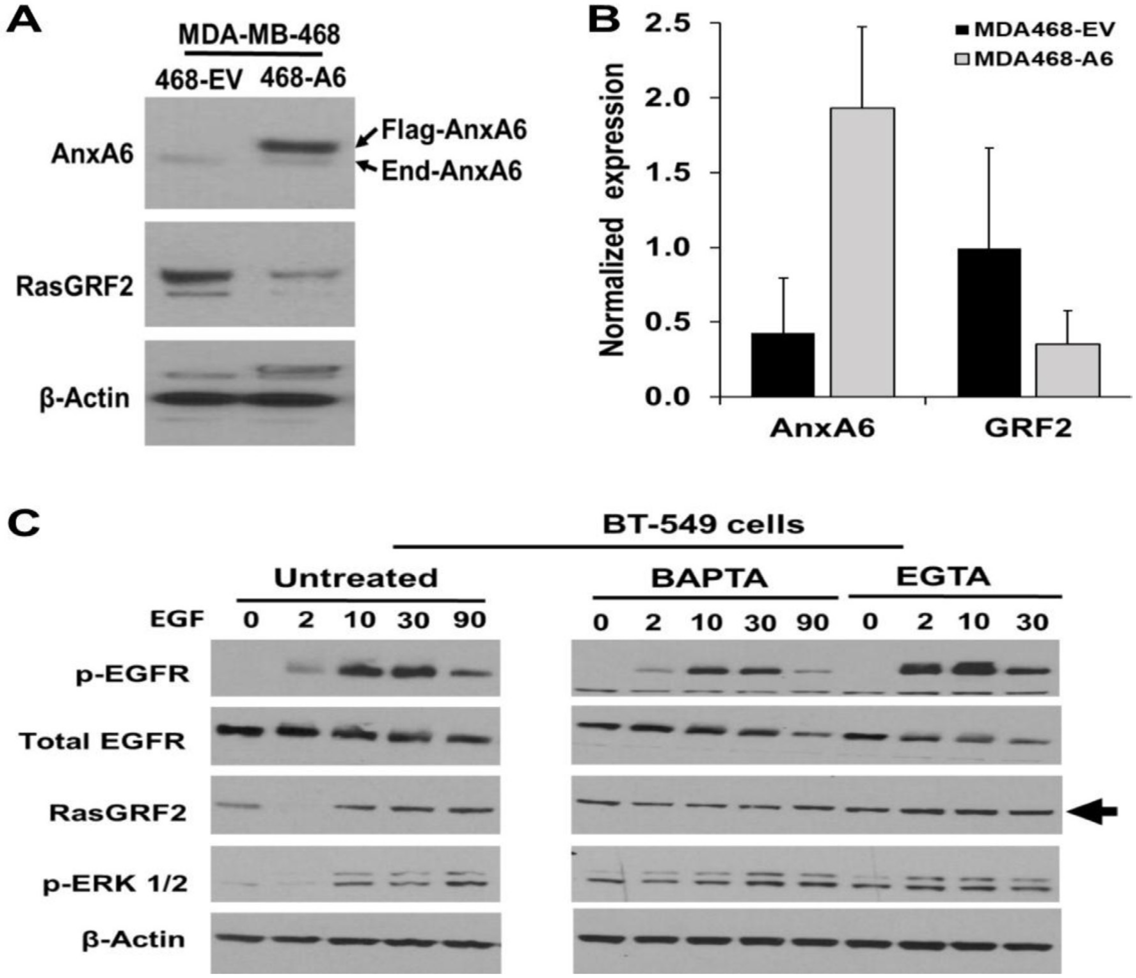

Supplementary Figure 5: Reciprocal expression of RasGRF2 and AnxA6 in MDA-MB-468 and effects of EGF stimulated Ca2+ influx on RasGRF2 in BT-549 cells. (**A** and **B**) Reciprocal cellular levels of AnxA6 and RasGRF2 in MDA-MB-468 basal-like TNBC Cells. Whole cell lysates from control (468-EV) and MDA-MB-468 cells stably expressing flag-tagged AnxA6 (468-A6) were analysed by western blotting (A) and the protein bands were quantified by densitometry using the NIH Image J software. Bars represent whole cellular levels of AnxA6 and RasGRF2 normalized to β-actin from three independent determinations (B). (**C**) Effect of EGFR activation and chelation of either intracellular or extracellular Ca2+ on the stability of RasGRF2 in the mesenchymal-like BT-549 breast cancer cells. Cells were treated with EGF with or without either BAPTA or EGTA for the indicated times. Equal amounts of whole cells extracts were analyzed by western blotting using antibodies to the indicated proteins. β-actin was used as the loading control. Arrow head indicates rapid down regulation of RasGRF2 within 2 min of EGF treatment in control cells (left panel) and the stabilization RasGRF2 following pretreatment of cell with EGTA (right panel).

Supplementary Table 1: Consistently up regulated genes following reduced expression of AnxA6

Accession no.	Gene ID	Gene description	BT-A6sh5 vs BT-A6sh2 a
**NM_006909**	RASGRF2	Ras protein-specific guanine nucleotide-releasing factor 2	15.319*
**ENST00000269141**	CDH2	Cadherin 2, type 1, N-cadherin (neuronal)	6.553*
**NM_032961**	PCDH10	Protocadherin 10	5.971*
**NM_032457**	PCDH7	Protocadherin 7	5.116*
**NM_000214**	JAG1	Jagged 1	4.742*
**ENST00000256104**	FABP4	Fatty acid binding protein 4, adipocyte	3.161*
**NR_039666**	MIR4461	MicroRNA 4461	2.796*
**BC004328**	SDHA	Succinate dehydrogenase complex, subunit A	1.086
**NM_001101**	ACTB	Actin, beta	0.815
**ENST00000361681**	ND6	NADH dehydrogenase, subunit 6 (complex I)	0.697
**NM_002852**	PTX3	Pentraxin 3, long	0.587
**NM_181353**	ID1	Inhibitor of DNA binding 1	0.548
**NM_130464**	NPIPL3	Nuclear pore complex interacting protein-like 3	0.307
**NR_002715**	RN7SL1	RNA, 7SL, cytoplasmic 1	–0.025
**AY014272**	POTEKP	POTE ankyrin domain family, member K, pseudogene	–0.141
**NM_021104**	RPL41	Ribosomal protein L41	–0.173
**ENST00000363841**	RN5S28	RNA, 5S ribosomal 28	–0.180
**NM_021009**	UBC	ubiquitin C	–0.186
**ENST00000390480**	TRAJ59	T cell receptor alpha joining 59	–0.194
**NM_000981**	RPL19	Ribosomal protein L19	–0.322
**ENST00000504154**	SLIT2	Slit homolog 2 (Drosophila)	–0.537

[a]The difference between fold up-regulation of genes in BT-A6sh5 versus control BT-EV cells and that in BT-A6sh2 versus control BT-EV cells. *indicates consistently up-regulated genes.

Supplementary Table 2: Consistently down-regulated genes following reduced expression of AnxA6

Accession no.	Gene ID	Gene description	BT-A6sh5 vs BT-A6sh2[a]
**NM_013230**	CD24	Signal transducer CD24 molecule	–9.555*
**NM_001098484**	SLC4A4	Solute carrier family 4 (sodium bicarbonate cotransporter), member 4	–6.564*
**ENST00000548553**	METTL7A	Methyltransferase like 7A	–4.920*
**NM_153000**	APCDD1	Adenomatosis polyposis coli down-regulated 1	–2.889*
**NM_006905**	PSG-1	Pregnancy specific beta-1-glycoprotein 1	–2.255*
**NM_001740**	CALB2	Calbindin 2	–1.718*
**ENST00000258733**	GPNMB	Glycoprotein (transmembrane) nonmetastatic melanoma protein B	–1.671*
**NM_178428**	LCE2A	Late cornified envelope 2A	0.505
**ENST00000341459**	SLC24A5	Solute Carrier Family 4 (Sodium Bicarbonate Cotransporter), Member 51	0.728
**NM_006520**	DYNLT3	Dynein Light Chain, T-Complex-Associated-Testis-Expressed Type 3	1.566
**NM_002982**	CCL2	Chemokine (C-C Motif) Ligand 2	6.771

[a]The difference between fold down-regulation of genes in BT-A6sh5 versus control BT-EV cells and that in BT-A6sh2 versus control BT-EV cells. *indicates consistently down-regulated genes.

Supplementary Table 3: TaqMan assays used in this study

Assay ID*	Gene ID	Gene assignment
Hs01086177_m1	FABP4	Fatty acid binding protein 4, adipocyte
Hs00204042_m1	METTL7A	Methyltransferase like 7A
Hs00537787_m1	APCDD1	Adenomatosis polyposis coli down-regulated 1
Hs00394798_m1	RASGFR2	Ras protein-specific guanine nucleotide-releasing factor 2
Hs01060665_g1	ACTB	Actin, beta
Hs03044178_g1	CD24	Cluster of Differentiation CD24 molecule
Hs00983056_m1	CDH2	Cadherin 2, type 1, N-cadherin (neuronal)
Hs01070032_m1	JAG1	Jagged 1
Hs00186798_m1	SLC4A4	Solute carrier family 4 (sodium bicarbonate cotransporter), member 4
Hs00418693_m1	CALB2	Calbindin 2
Hs00361541_g1	CSPG4	Chondroitin sulfate proteoglycan 4
Hs02341873_m1	ST8SIA6	ST8 alpha-N-acetyl-neuraminide alpha-2,8-sialyltransferase 6

*Each assay comprises a pair of gene specific primers and a TaqMan probe.
